# Introducing Mediterranean Lupins in Lambs’ Diets: Effects on Growth and Digestibility

**DOI:** 10.3390/ani11040942

**Published:** 2021-03-26

**Authors:** Mariana Almeida, Sofia Garcia-Santos, Ana Nunes, Sara Rito, Jorge Azevedo, Cristina Guedes, Severiano Silva, Luís Ferreira

**Affiliations:** 1 Veterinary and Animal Research Centre (CECAV), University of Trás-os-Montes e Alto Douro, Quinta de Prados, 5000-801 Vila Real, Portugal; jazevedo@utad.pt (J.A.); cguedes@utad.pt (C.G.); ssilva@utad.pt (S.S.); 2 Department of Zootechnics, University of Trás-os-Montes e Alto Douro, Quinta de Prados, 5000-801 Vila Real, Portugal; ssantos@utad.pt (S.G.-S.); ananuness94@hotmail.com (A.N.); lmf@utad.pt (L.F.); 3 Centre for the Research and Technology Agro-Environmental and Biological Sciences (CITAB), University of Trás-os-Montes e Alto Douro, Quinta de Prados, 5000-801 Vila Real, Portugal; 4 Instituto Superior de Agronomia, School of Agriculture, University of Lisbon, Tapada da Ajuda, 1349-017 Lisbon, Portugal; sara_rito@hotmail.com

**Keywords:** lambs, lupins, growth performance, digestibility

## Abstract

**Simple Summary:**

Finding alternative protein sources has been one of the most important issues in the animal nutrition field in the last decades. Due to its chemical composition and previously reported positive results on lambs’ performance and digestibility, lupins were the main focus of this study. Data were collected from two distinct trials with seven different diets tested in total. The chemical composition of the raw materials was analyzed, as well as total dry matter, hay dry matter, and crude protein intake. Lambs’ growth was accompanied throughout the trials and their performance was measured. Dry matter, organic matter, and NDF digestibility of each diet was also determined. Low incorporations of lupins had no impact on performance and digestibility of any of the studied fractions. The highest tested incorporation had a negative impact on the overall values of feed intake and the NDF digestibility. The results of this study suggest that low inclusions might have no impact on lambs’ growth, intake, and digestibility.

**Abstract:**

Lupins are suitable candidates to replace soybean meal in livestock feeding in the Mediterranean area, presenting a solution for the European Union’s dependence on soybean importations. This study aimed to assess the effect of incorporating *Lupinus albus* and *Lupinus luteus* into *Churra da Terra Quente* lambs’ diets on growth performance and digestibility. Two trials were conducted over two years. In trial 1, two experimental diets containing 50 g/kg *Lupinus albus* and 50 g/kg *Lupinus luteus* were tested. In trial 2, lambs were fed with diets containing higher incorporations of *Lupinus luteus* (100, 150, and 200 g/kg: LL10, LL15, and LL20, respectively). Total dry matter, hay dry matter, and crude protein intake were calculated, as well as average daily gains. At the end of the growth trials, dry matter, organic matter, and NDF digestibility was determined. Incorporating 50 g/kg of lupins did not affect (*p* > 0.05) the performance. Lambs fed on LL20 diets presented the lowest HDMI and CPI values (*p* < 0.05). The highest intakes (*p* < 0.05) were observed from LL15 lambs. No differences were found in apparent digestibility coefficients between diets (*p* > 0.05), except for NDF digestibility which was highest (*p* < 0.05) for LL20. The optimum level of lupin inclusion in lambs’ diets seems to be 150 g/kg.

## 1. Introduction

Research in the last decades has been focused on creating solutions and providing alternative protein sources to animal feeding diminishing the European Union (EU) dependence on importing soybean [1]. Overall, lupin grains have only a slightly lower protein content (350–400 g/kg DM) [2] compared to soybean meal (~440 g/kg DM) [3] and are very well adapted to the Mediterranean conditions, being ideal for low-input cropping systems and crop rotation [1,3]. This makes them suitable candidates to partially or totally replace soybean meal in livestock feeding. Although the introduction of these legume grains into livestock diets has been discussed in the last decades [4,5], recently, it gained higher visibility due to an increasing awareness from the consumers towards the provenience of animal feed [6,7]. The introduction of white (*Lupinus albus*) and narrow-leafed lupin (*Lupinus angustifolius*) in ruminant feeding, for example, has been widely studied over the years [8,9] also in in vitro studies [10]. Besides the positive results found on growth performance [11,12,13], lupin incorporation in sheep feeds has been reported to be an appropriate supplement to increase reproductive performance [14,15]. Although traditionally used for grazing sheep in Portugal and other Mediterranean countries [16], few studies have tested the incorporation of yellow lupin (*Lupinus luteus*) on ruminant feeding. Sheep native breeds, associated with pasture systems, are seen as a solution for reducing livestock emissions [17], and so it becomes even more relevant to study different feeding alternatives.

This study aimed to assess the effect of the inclusion of *Lupinus albus* and *Lupinus luteus* grains into *Churra da Terra Quente* lambs’ diets’ effect on growth performance and digestibility.

## 2. Materials and Methods

Trial 1 was performed to better understand the effect of introducing small percentages of *Lupinus albus* and *Lupinus luteus* in sheep feeding. Based on these results, a second trial was designed to evaluate the effect of increasing the percentage of *Lupinus luteus* in diets on lamb’s growth performance and digestibility. Both trials were conducted in the animal facilities of the University of Trás-os-Montes and Alto Douro (UTAD) at Vila Real (Portugal), and the animals were handled according to the Portuguese law on animal welfare in experimental research [18]. The protocol was approved by the ORBEA (Animal Welfare Body) of the University Trás-os-Montes and Alto Douro (669-e-DZ-2018).

### 2.1. Animals and Housing

The two trials were performed on a total of 28 *Churra da Terra Quente* male lambs. In trial 1, twelve lambs with initial body weight (BW) of 18 ± 2.8 kg and ages between 92 and 110 days were divided into three groups of four animals each. In trial 2, sixteen lambs with initial body weight (BW) of 16 ± 2.59 kg and ages between 92 and 110 days were divided into four groups of four animals each. In both trials, the animals were randomly assigned to the groups. All animals were kept in group pens (2 × 4 m each pen) for the entire duration of the growth trials, with access to a feeder per lamb (40 cm width) and a water cup per pen (24 cm diameter). During the trials, ambient temperature and humidity were controlled (20 ± 3 °C, 50 ± 5%, respectively), and the animals were exposed to a natural lighting cycle (11 h light/13 h dark).

### 2.2. Diets

In trial 1, each group was provided with a different diet: A control diet (C, 150 g/kg soybean meal) with no lupin incorporation, a diet with 50 g/kg *Lupinus luteus* cv. Mister (LL5) and a diet with 50 g/kg *Lupinus albus* cv. Nacional (LA5) (Table 1). The same experimental design was used in trial 2: One group was provided with a control diet (C, 150 g/kg soybean meal) and the other three groups were fed with a different level of *Lupinus luteus* cv. Mister (100 g/kg, 150 g/kg, 200 g/kg; LL10, LL15, LL20, respectively) (Table 1).

White (*Lupinus albus* cv. Nacional) and yellow lupin (*Lupinus luteus* cv. Mister) grains, wheat, and soybean meal were acquired from a local company and analyzed for their chemical composition (Table 2). Lamb growth requirements were calculated according to AFRC [19] recommendations. Diets were formulated to be isonitrogenous (11–13% CP) and isoenergetic (9 MJ kg^−1^ DM), according to Kirchgessner and Kellner [20]. The animals had an adaptation period of 21 days before each trial. After that, the ingested quantities were adjusted to the lambs’ growth requirements. The concentrate feeds were weighed, provided, and controlled daily and individually. The lambs had access to meadow hay and water ad libitum during both trials. Meadow hay was milled roughly around 4 cm and lupines and wheat were milled at 4 mm. Soybean meal was presented with commercial granulometry. Samples of dietary components were collected once every fifteen days for chemical analysis. The feed refusals were weighed in the morning and then placed in labeled plastic bags. They were subsequently frozen at −15 °C. At the end of the experimental period, the samples were thawed, homogenized, dried at 55 °C for 72 h, and then ground to pass through a 1-mm screen (Tecnal, Piracicaba, São Paulo, Brazil). The ground samples were then stored in properly sealed, airtight, plastic containers (ASS, Ribeirão Preto, São Paulo, Brazil) until needed for laboratory analysis.

### 2.3. Growth Trials

Before the beginning of the experiments, the lambs had an adaptation period of 21 days in which they were gradually introduced to the pens and diets. Animals were weighed using a scale (Kern HUS 150K50, KERN & Sohn GmbH, Balingen, Germany) at the beginning of the trial and then weekly, always before the first meal of the day, and average daily gains (ADG) were calculated considering the differences of weight between each week for each lamb. Feed conversion ratios (FCR) were obtained by dividing the daily hay and concentrate intake by the ADG. Concentrate and hay refusal data were collected daily. Since hay was offered ad libitum to each group, hay intake was estimated for each lamb each day based on their refusals and hay dry matter intake (HDMI) was calculated. Total dry matter intake (DMI), neutral detergent fiber intake (NDFI), and crude protein intake (CPI) were calculated based on the estimated hay intake and individual concentrate intake. Both trials lasted 12–16 weeks and finished as soon as all the lambs achieved a BW of approximately 25–27 kg.

### 2.4. Digestibility Trials

At the end of the growth trials, all the animals were transferred to individual metabolic cages. The lambs were fed once a day with the same diets they had previously been receiving, restricted to their maintenance levels [19] (Table 3). Daily intakes were controlled, and feed refusals were registered daily. After an adaptation period of 5 days, feces were collected for 7 days. Feces were collected daily, weighed, and stored at −17 °C in plastic containers. After homogenization, two samples from each lamb were collected and dried at 70 °C for 48 h. Samples were milled and analyzed for dry matter, organic matter, and NDF. Dry matter, organic matter, and NDF apparent digestibilities were obtained using the general formula presented by Mcdonald et al. [21]:

$$\frac{\mathrm{Fraction}\;\mathrm{consumed}\;-\;\mathrm{Fraction}\;\mathrm{in}\;\mathrm{feces}}{\mathrm{Fraction}\;\mathrm{consumed}}\;\times \;100$$

### 2.5. Chemical Composition

Samples from all the diet components and feces were milled at 1 and 0.5 mm (Retsch cutting mill, model SM1, Retsch GmbH, Haan, Germany) and analyzed through AOAC [22] procedures for dry matter (DM) (934.01), organic matter (OM) (942.05), ether extract (EE) (920.39), crude protein (CP) (954.01), and starch (996.11). A Velp Scientifica apparatus (Velp Scientifica srl, Usmate, Italy) was used to determine Kjedhal N, which was then multiplied by a conversion factor of 6.25 to determine CP content. A solvent recovery extractor for oils and fats (JP Selecta, Det-Gras N, Barcelona, Spain) was used to determine EE content. To determine starch content, an enzymatic kit K-TSTA-100A (Megazyme, Enterprise Ireland, Dublin, Ireland) and a spectrometer UVmin-1240 (Shimadzu, Quioto, Japan) were used. Neutral detergent fiber (NDF) and acid detergent fiber (ADF) were obtained through the Robertson and Van Soest and Van Soest et al. [23,24] methodologies. Acid detergent lignin (ADL) was determined by solubilization of cellulose with sulfuric acid [23]. Soluble sugar content was determined using a UV-VIS spectrophotometry UVmin-1240 (Shimadzu, Quioto, Japan; 625 nm) through the anthrone methodology [25].

### 2.6. Statistical Analyses

Statistical analysis was performed on IBM SPSS^®^, version 27 [26]. Prior to analysis, data were checked for normality and homogeneity using Shapiro–Wilk and Levene tests. Since results did not satisfy the assumptions of normality and transformations were ineffective, data from the growth trials were then subjected to a Friedman test. A Kruskal–Wallis test was performed on digestibility data. Whenever significant differences were found, the data were subjected to a pair-wise Wilcoxon–Mann–Whitney test to establish levels of difference. Diets were used as the main factor, and significance was declared at *p* < 0.05. Despite not satisfying the assumptions of normality, data from the growth trial in trial 2 were tested for linear and quadratic contrasts since different levels of inclusion were being compared.

## 3. Results

### 3.1. Growth Trials

No palatability problems were observed in all the treatments involving lupin incorporation during both trials. The inclusion of small percentages of lupins had no effect (*p* > 0.05) on intake or growth, as is shown by the results of trial 1 (Table 4).

Higher inclusions of *Lupinus luteus* and total replacement of soybean meal had no impact (*p* > 0.05) on growth performance (Table 5). ADG values were comparable between C groups of both trials. Lambs from group LL20 presented the lowest HDMI values (*p* < 0.05). Concerning protein intake (CPI), there were similarities between groups C and LL15, which presented superior values (*p* < 0.05) than LL10 and LL20. Hay ingestion (HDMI) was around 60 g/day lower (*p* < 0.05) for LL20 when compared to C and LL15 groups. These groups presented the highest HDMI values. There seems to be no linear or quadratic trend in most of the data from the growth trials (*p* > 0.05), except for HDMI (*p* = 0.029) and CPI (*p* = 0.016), which show a linear trend.

### 3.2. Digestibility Trials

One lamb from trial 1 died before the beginning of digestibility trials, so only three animals from C group remained. In trial 1, no differences were found in apparent digestibility coefficients (Table 6). Lambs from LL15 and LL20 presented the highest NDFD digestibility (659.15 and 661.45 g/kg, respectively; Table 7).

## 4. Discussion

As expected, lupins used in this study presented high levels of CP, as well as fiber fraction values, and negligible amounts of starch, as described by other authors [27,28], although other authors [28] have reported lower CP values (362 g/kg DM) for *Lupinus luteus* cv. Mister. The results obtained in the first trial suggest that low inclusions of both lupin species (50 g/kg as fed) have no effect (*p* > 0.05) on any of the studied parameters. These results are consistent with those observed in previous studies, which have reported no differences (*p* > 0.05) in ADG, intakes, and feed efficiency in lambs [11,12,13,29] with lupin inclusion to their feed. Fychan et al. [30] observed no differences (*p* > 0.05) in ADG of Suffolk castrated lambs fed 23% narrow-leafed lupin (*Lupinus angustifolius*) and 16% yellow lupin (*Lupinus luteus*), which is consistent with our results in both trials. However, other studies have reported the effects of higher inclusion of lupin seeds on ADG [11,12,31]. Lower than desirable amounts of methionine in lupins paired with the fact that low proportions of these amino acids bypass rumen degradation to reach the small intestine could affect ruminant’s performance [4,13], especially at high levels of feeding, which might explain lower values of ADG if diets only adjusted to be isonitrogenous and not for amino acid profile.

Overall, increasing lupin inclusion levels in lambs’ diets led to a reduced HDMI (*p* < 0.05), around 32–63 g/day less than the other groups. Since lupins present higher fiber levels than soybean meal, lambs from LL20 may have compensated for this with reduced hay intake. In a study by Lestingi et al. [31] with Gentile di Puglia male lambs, the inclusion of 250 g/kg of *Lupinus albus* cv. Multitalia significantly reduced (*p* < 0.01) the average daily feed intake when compared to another faba bean (*Vicia faba* L.) and peas (*Pisum sativum* L.) (690 vs. 740 and 770 g/day). In contrast, White et al. [12] have reported no statistical differences (*p* > 0.01) between 35% and 70% lupin inclusion on the diets, although DMI reduced with increased incorporations (1123 and 1056 g/day, respectively). Our study presents lower (*p* < 0.05) HDMI intakes for LL20 with no effect (*p* > 0.05) on lambs’ performance. Antinutritional components, such as alkaloids, which have a negative effect on palatability and therefore on ingestion [4,28,32], were present at a very low level. Considering these levels and the fact that the lambs showed no aversion when provided the concentrate feed, consuming all of it every day, it is safe to assume that the scarce presence of bitter components did not reduce intakes.

As happened with performance traits, digestibility seems to suffer no alterations with small lupin inclusions in the diet. However, the two highest lupin inclusions in this study (LL15 and LL20) had an effect (*p* < 0.05) on NDF digestibility, which was higher at these levels. Higher inclusions of lupin grains (75% vs. 35%) in Merino weaner sheep diets have provided similar results, with an increase (*p* < 0.01) of OMD proportional to the level of lupins in the diet [12]. Ephrem et al. [33] reported higher DM and OM apparent digestibility (*p* < 0.05) in Washera lambs supplemented with sweet lupin grains, between 682 and 715 g/kg. Higher intakes generally result in increases in the passage rate of digesta, due to a lower exposure to digestive enzymes [21], however, in this study, there was no effect (*p* > 0.05) of diet on DMD and OMD.

## 5. Conclusions

The inclusion of lupins in diets of growing lambs had no significant effect on growth performance and digestibility, although there was an effect on forage intake. We hypothesize that 150 g/kg inclusion of lupins in the diet may be an optimum value since it presented the best results, especially considering the aim of this study was maximum soybean meal replacement. Total replacement of soybean meal resulted in worse intake results due to reduced hay intake, but it did not affect performance.

Overall, lupins seem to be a possible solution to replace soybean meal in lambs grown in the Mediterranean region. Ideally, higher incorporations would need to be studied, as well as a higher number of animals, to draw further conclusions.

## Figures and Tables

**Table 1:** Levels of components (g/kg as fed) and chemical composition of the ingested diets (g/kg DM) of growing lambs.

Diets	Diet Components					Chemical Composition		
	Soybean Meal	Wheat	*Lupinus albus*	*Lupinus luteus*	Hay	DM	CP	NDF
Trial 1								
C	150	20	0	0	830	930	121	637
LL5	100	20	0	50	830	933	117	629
LA5	100	20	50	0	830	923	114	632
Trial 2								
C	170	20	0	0	810	929	130	626
LL10	100	20	0	100	780	925	127	621
LL15	50	20	0	150	780	937	128	628
LL20	0	20	0	200	780	941	128	628

C—Control, LL5—*Lupinus luteus* 50 g/kg, LA5—*Lupinus albus* 50 g/kg, LL10—*Lupinus luteus* 100 g/kg, LL15—*Lupinus luteus* 150 g/kg, LL20—*Lupinus luteus* 200 g/kg, DM—Dry Matter, CP—Crude Protein, NDF—Neutral Detergent Fiber.

**Table 2:** Chemical composition of diet components (g/kg DM) used in both trials.

Raw Materials	Chemical Composition								
	DM	OM	CP	EE	NDF	ADF	ADL	Soluble Sugars	Starch
*Lupinus albus*	954	963	347	75.5	220	181	13.9	50.1	1.7
*Lupinus luteus*	954	954	431	40.2	257	199	9.7	40.6	7.2
Soybean meal (Trial 1)	888	930	510	14.5	162	82.7	4.0	98.6	10.5
Soybean meal (Trial 2)	887	929	510	13.8	161	82.6	3.1	96.2	9.9
Wheat	867	984	109	18.2	128	29.3	6.4	4	640
Hay (Trial 1)	939	931	51.0	7.7	736	504	75.2	9.4	8.0
Hay (Trial 2)	929	921	50.8	7.1	730	465	72.4	9.1	7.6

DM—Dry Matter, OM—Organic matter, CP—Crude Protein, EE—Ether Extract, NDF—Neutral Detergent Fiber, ADF—Acid Detergent Fiber, ADL—Acid Detergent Lignin.

**Table 3:** Levels of components (g/kg as fed) ingested during the digestibility trials.

Diets	Chemical Composition				
	Soybean Meal	Wheat	*Lupinus albus*	*Lupinus luteus*	Hay
Trial 1					
C	125	0	0	0	500
LL5	90	0	0	40	500
LA5	105	0	30	0	500
Trial 2					
C	70	10	0	0	500
LL10	30	10	0	30	500
LL15	15	10	0	45	500
LL20	0	10	0	90	500

C—Control, LL5—*Lupinus luteus* 50 g/kg, LA5—*Lupinus albus* 50 g/kg, LL10—*Lupinus luteus* 100 g/kg, LL15—*Lupinus luteus* 150 g/kg, LL20—*Lupinus luteus* 200 g/kg.

**Table 4:** Intake and growth performance (g/day) of growing lambs in trial 1.

Parameters	Diets			SEM	*p*
	C (*n* = 4)	LL5 (*n* = 4)	LA5 (*n* = 4)		
ADG	125	151	122	15.1	0.936
FCR	5.8	5.0	6.1	0.59	0.825
DMI	702	710	717	12.1	0.682
HDMI	566	566	569	11.3	0.682
NDFI	436	443	444	8.5	0.392
CPI	91.0	91.6	90.9	1.23	0.682

C—Control, LL5—*Lupinus luteus* 50 g/kg, LA5—*Lupinus albus* 50 g/kg, ADG—Average Daily Gain, FCR—Feed Conversion Rate, DMI—Total Dry Matter Intake, HayDMI—Hay Dry Matter Intake, NDFI—Neutral Detergent Fiber Intake, CPI—Crude Protein Intake, SEM—standard error of the mean.

**Table 5:** Intake and growth performance (g/day) of growing lambs in trial 2.

Parameters	Diets				SEM	*p*
	C (*n* = 4)	LL10 (*n* = 4)	LL15 (*n* = 4)	LL20 (*n* = 4)		
ADG	116	96.0	104	95.2	7.6	0.198
FCR	6.5	7.5	7.4	7.4	0.60	0.145
DMI	735 ^a,b^	705 ^b^	755 ^a^	690 ^b^	14.6	0.019
HDMI	556 ^a^	526 ^a^	557 ^a^	493 ^b^	14.0	0.019
NDFI	441 ^a,b^	423 ^b^	460 ^a^	409 ^b^	10.4	0.038
CPI	108 ^a^	104 ^b^	108 ^a^	104 ^b^	0.9	0.019

C—Control, LL10—*Lupinus luteus* 100 g/kg, LL15—*Lupinus luteus* 150 g/kg, LL20—*Lupinus luteus* 200 g/kg, ADG—Average Daily Gain, FCR—Feed Conversion Rate, DMI—Total Dry Matter Intake, HDMI—Hay Dry Matter Intake, NDFI—Neutral Detergent Fiber Intake, CPI—Crude Protein Intake, SEM—standard error of the mean. Different superscript letters (a,b) on the same row indicate significant differences (*p* < 0.05).

**Table 6:** Apparent digestibility coefficients (g/kg) of growing lambs in trial 1.

Digestibility	Diets			SEM	*p*
	C (*n* = 3)	LL5 (*n* = 4)	LA5 (*n* = 4)		
DMD	692	648	656	32.9	0.591
OMD	705	664	675	30.1	0.565
NDFD	686	626	638	24.4	0.149

C—Control, LL5—*Lupinus luteus* 50 g/kg, LA5—*Lupinus albus* 50 g/kg, DMD—Dry Mater Apparent Digestibility, OMD—Organic Matter Apparent Digestibility, NDFD—Neutral Detergent Fiber Apparent Digestibility, SEM—standard error of the mean.

**Table 7:** Apparent digestibility coefficients (g/kg) of growing lambs in trial 2.

Digestibility	Diets				SEM	*p*
	C (*n* = 4)	LL10 (*n* = 4)	LL15 (*n* = 4)	LL20 (*n* = 4)		
DMD	640	649	661	677	10.5	0.228
OMD	658	663	682	694	10.0	0.087
NDFD	624 ^b^	632 ^b^	659 ^a^	662 ^a^	10.5	0.037

C—Control, LL10—*Lupinus luteus* 100 g/kg, LL15—*Lupinus luteus* 150 g/kg, LL20—*Lupinus luteus* 200 g/kg, DMD—Dry Mater Apparent Digestibility, OMD—Organic Matter Apparent Digestibility, NDFD—Neutral Detergent Fiber Apparent Digestibility, SEM—standard error of the mean. Different superscript letters (a,b) on the same row indicate significant differences (*p* < 0.05).

## Data Availability

The data presented in this study are available on request from the corresponding author. The data are not publicly available to preserve privacy of the data.
